# Grassland vertical height heterogeneity predicts flower and bee diversity: an UAV photogrammetric approach

**DOI:** 10.1038/s41598-023-50308-9

**Published:** 2024-01-08

**Authors:** Michele Torresani, Duccio Rocchini, Giada Ceola, Jan Peter Reinier de Vries, Hannes Feilhauer, Vítězslav Moudrý, Harm Bartholomeus, Michela Perrone, Matteo Anderle, Hannes Andres Gamper, Ludovico Chieffallo, Enrico Guatelli, Roberto Cazzolla Gatti, David Kleijn

**Affiliations:** 1 Faculty of Agricultural, Environmental and Food Sciences, Free University of Bolzano/Bozen, Piazza Universitá/Universitätsplatz 1, 39100 Bolzano/Bozen, Italy; 2 BIOME Lab, Department of Biological, Geological and Environmental Sciences, Alma Mater Studiorum University of Bologna, via Irnerio 42, 40126 Bologna, Italy; 3 Department of Spatial Sciences, Faculty of Environmental Sciences, Czech University of Life Sciences Prague, Kamýcka 129, Praha - Suchdol, 16500 Czech Republic; 4 Plant Ecology and Nature Conservation Group, Wageningen University, Droevendaalsesteeg 3a, Wageningen, 6708PB The Netherlands; 5 Remote Sensing Centre for Earth System Research (RSC4Earth), Leipzig University, Leipzig, Germany; 6 German Centre for Integrative Biodiversity Research (iDiv) Halle-Jena-Leipzig, Leipzig, Germany; 7 Department of Remote Sensing, Helmholtz-Centre for Environmental Research - UFZ, Permoserstr. 15, 04318 Leipzig, Germany; 8 Laboratory of Geo-Information Science and Remote Sensing, Wageningen University and Research, P.O. Box 47, 6700 AA Wageningen, The Netherlands; 9 Eurac Research, Inst. for Alpine Environment, Bolzano, Italy; 10 Department of Environmental Science and Policy, University of Milan, Milan, Italy; 11 Visual art, FEIMC, Bolzano, Italy

**Keywords:** Biodiversity, Environmental impact, Ecosystem services, Grassland ecology

## Abstract

The ecosystem services offered by pollinators are vital for supporting agriculture and ecosystem functioning, with bees standing out as especially valuable contributors among these insects. Threats such as habitat fragmentation, intensive agriculture, and climate change are contributing to the decline of natural bee populations. Remote sensing could be a useful tool to identify sites of high diversity before investing into more expensive field survey. In this study, the ability of Unoccupied Aerial Vehicles (UAV) images to estimate biodiversity at a local scale has been assessed while testing the concept of the Height Variation Hypothesis (HVH). This hypothesis states that the higher the vegetation height heterogeneity (HH) measured by remote sensing information, the higher the vegetation vertical complexity and the associated species diversity. In this study, the concept has been further developed to understand if vegetation HH can also be considered a proxy for bee diversity and abundance. We tested this approach in 30 grasslands in the South of the Netherlands, where an intensive field data campaign (collection of flower and bee diversity and abundance) was carried out in 2021, along with a UAV campaign (collection of true color-RGB-images at high spatial resolution). Canopy Height Models (CHM) of the grasslands were derived using the photogrammetry technique “Structure from Motion” (SfM) with horizontal resolution (spatial) of 10 cm, 25 cm, and 50 cm. The accuracy of the CHM derived from UAV photogrammetry was assessed by comparing them through linear regression against local CHM LiDAR (Light Detection and Ranging) data derived from an Airborne Laser Scanner campaign completed in 2020/2021, yielding an $R^2$ of 0.71. Subsequently, the HH assessed on the CHMs at the three spatial resolutions, using four different heterogeneity indices (Rao’s Q, Coefficient of Variation, Berger–Parker index, and Simpson’s D index), was correlated with the ground-based flower and bee diversity and bee abundance data. The Rao’s Q index was the most effective heterogeneity index, reaching high correlations with the ground-based data (0.44 for flower diversity, 0.47 for bee diversity, and 0.34 for bee abundance). Interestingly, the correlations were not significantly influenced by the spatial resolution of the CHM derived from UAV photogrammetry. Our results suggest that vegetation height heterogeneity can be used as a proxy for large-scale, standardized, and cost-effective inference of flower diversity and habitat quality for bees.

## Introduction

In the last decades, we have witnessed a decrease in plant and insect biodiversity in agricultural landscapes, resulting in the loss of benefits for crops and humans^28,31^. The causes of this can be found in changes of land use causing habitat loss and fragmentation^27,69,76^, increasingly intensive agriculture, and climate change^71^. All these factors have affected the presence of particular niches for different types of insects^31^. Yet insect pollinators are essential for the maintenance of wild plant species, contributing to cultural ecosystem services and agricultural yields^6,18^. They play a crucial role in the long-term sustainability of plant communities, and their loss can lead to a decline in plant diversity, altering vegetation composition^84^. The economic value of insect pollinators is immense, with estimates suggesting that they contribute to global food production worth more than 150 billion euros per year^20,21,54^. Therefore, insect pollinators are essential for maintaining the health and productivity of both agricultural and natural ecosystems, as well as for ensuring a continued provisioning of ecosystem services^30^.

Earth observation and remote sensing data have become valuable tools for estimating different aspects of biodiversity worldwide^63,65^. Significant advancements in sensor technology (with increased spatial and spectral resolution) and vectors (able to cover large areas with higher revisit frequency) have made remote sensing rapid and cost-effective to obtain extensive environmental data at various temporal and spatial scales^7^. Over the past few years, there has been a development of different methods and techniques utilizing remote sensing data to assess biodiversity at various spatial levels^7^. Some of these approaches rely on indirect associations between the variability of remotely sensed information and species diversity^81,82^. Notably, recent investigations have specifically concentrated on exploring the link between LiDAR data and species diversity. This approach, called “Height Variation Hypothesis” (HVH), states that, in a considered ecosystem, the higher the vegetation height heterogeneity (HH) assessed by LiDAR information, the higher the availability of different niches that can host more diverse species. Vertical vegetation structure, which encompasses aspects of habitat heterogeneity, plays a critical role in supporting biodiversity. It is considered one of the drivers of biodiversity, directly influencing species distribution and diversity, population dynamics, and ecological interactions^41^. By providing a variety of microhabitats and vertical niches, the vertical vegetation structure offers opportunities for different species to find suitable habitats and resources, promoting species coexistence and enhancing overall biodiversity. It contributes to ecosystem stability and resilience, making it a key component in conservation and management efforts aimed at preserving and enhancing biodiversity in various ecosystems^26^. Torresani et al.^79,80^ tested this approach positively in different forested areas using both Airborne Laser Scanning (ALS, where the LiDAR sensor is mounted on an aircraft) and space-borne GEDI (Global Ecosystem Dynamics Investigation) LiDAR data^14,16,34,53^ for the assessment of tree species diversity. Tamburlin et al.^72^ also tested the methodology in forested areas using ALS data, showing that the Canopy Height Model (CHM) is the most appropriate LiDAR metric for an accurate estimation of vegetation height heterogeneity and inference of species diversity. The approach has been used not only to assess vegetation diversity but also to estimate animal diversity, different studies showed that the variability in habitat structure has a significant effect on the bird diversity in both agricultural and forest ecosystems^2,43^. However, there is limited research specifically on the correlation between vegetation structure and insect diversity, particularly at a fine scale observed in grasslands.

In this paper, we aim to test this approach in a grassland ecosystem to understand if the vegetation grassland HH assessed through remote sensing techniques can be considered a proxy for flower diversity and subsequently for bee diversity and abundance. As grassland vegetation structures occur at very fine spatial scales, there is a need for structural information at a very high spatial resolution. While there have been a few studies^40^ exploring the use of LiDAR for grassland characterization, the limited available evidence introduces uncertainty regarding its effectiveness in this context. Furthermore, while ALS data depend on a dedicated aircraft campaign and may involve higher costs, operational testing of our hypothesis on Unoccupied Aerial Vehicles (UAVs) data might provide a practical and scalable approach. The recently developed technology centered around these new vectors, specifically photogrammetry that employs structure-from-motion algorithms, has resulted in the creation of highly precise orthomosaics and 3D information across vast areas at a relatively low expense, with spatial resolutions ranging from centimeters to millimeters suitable to derive information on vegetation structure^1^. Previous researches^9,10,33,38,77^ has demonstrated that UAV imagery can be utilized to gauge vegetation attributes, including diversity, species, and plant species distribution, as well as to map and track invasive species. In this context, our prior study^77^ established, in the same study area, a positive correlation between flower cover, estimated through UAV images, and bee diversity, further emphasizing the versatility of UAV technology in understanding and quantifying key ecological relationships.

**Figure 1:**
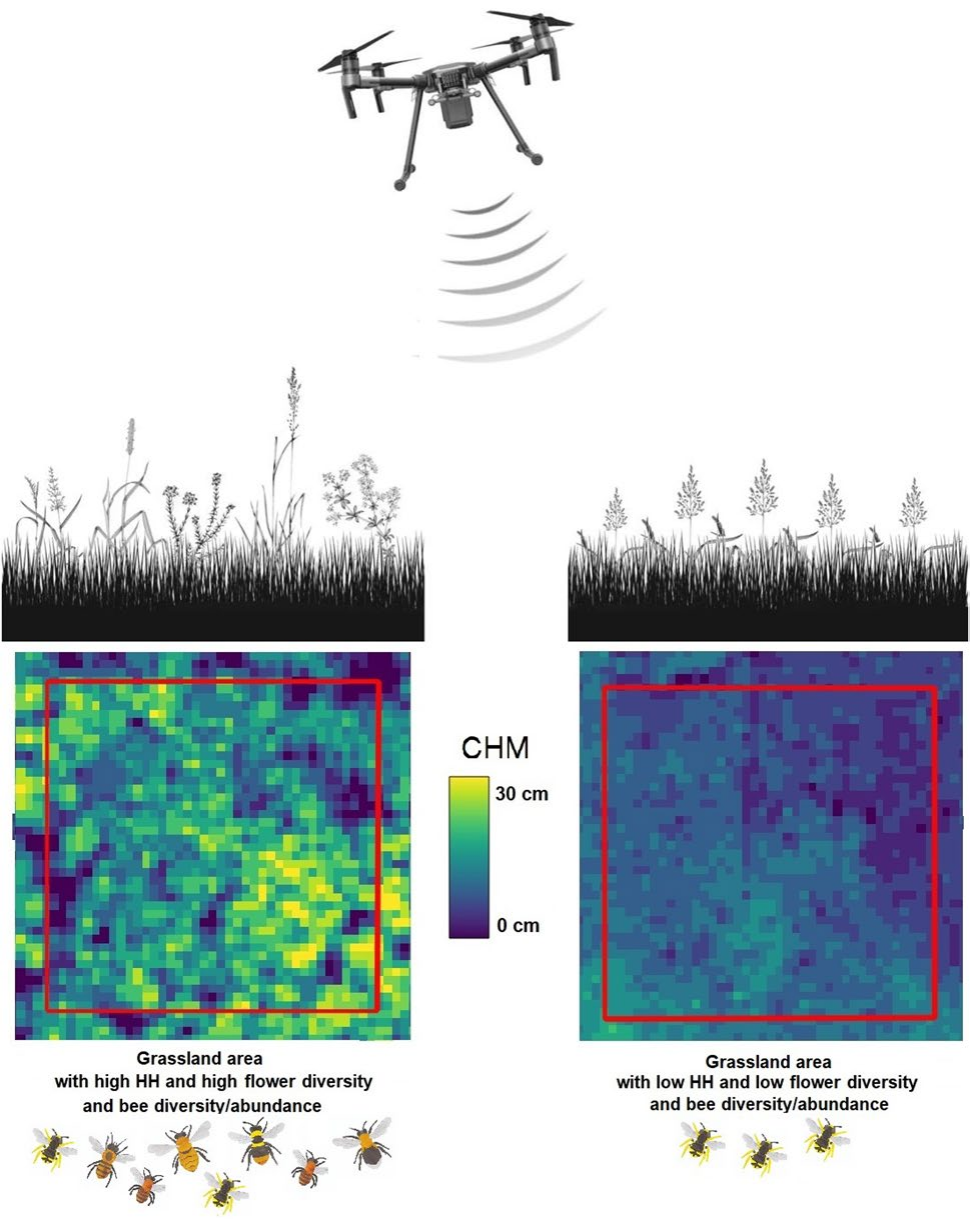
A graphical summary of the main expectations of this study. Grassland ecosystems with high HH (assessed through CHM derived by UAV photogrammetric images) with a complex vertical structure (seen from the side in the upper figure and from above in the lower figure) and high environmental heterogeneity are expected to have a high flower diversity and high bee diversity and abundance (figure on the left). On the other hand, grassland areas with low HH might have lower flower diversity and bee diversity and abundance (figure on the right).

The aim of this study is to test whether we can estimate flower diversity and bee abundance and diversity by testing the Height Variation Hypothesis in as highly dense and fine structured ecosystem such as grasslands by using 3D information derived with photogrammetric analysis using UAV RGB (true-colored) images at high spatial resolution (Fig. 1). Specifically, we assessed the HH with four different heterogeneity indices (Rao’s Q, Coefficient of Variation—CV—, Berger–Parker index and Simpson’s D index) using CHM data derived from UAV photogrammetric analysis previously validated with local ALS data. Successively, we correlated the derived HH with field-derived flower and bee diversity (species richness) and abundance. Finally, we investigated the influence of varying spatial resolutions (10 cm, 25 cm, and 50 cm) on the observed relationships. Our study focuses on grasslands located in the southeastern region of the Netherlands, which exhibit a range of management intensities, resulting in varying degrees of flower cover.

## Materials and methods

### Study areas

**Figure 2:**
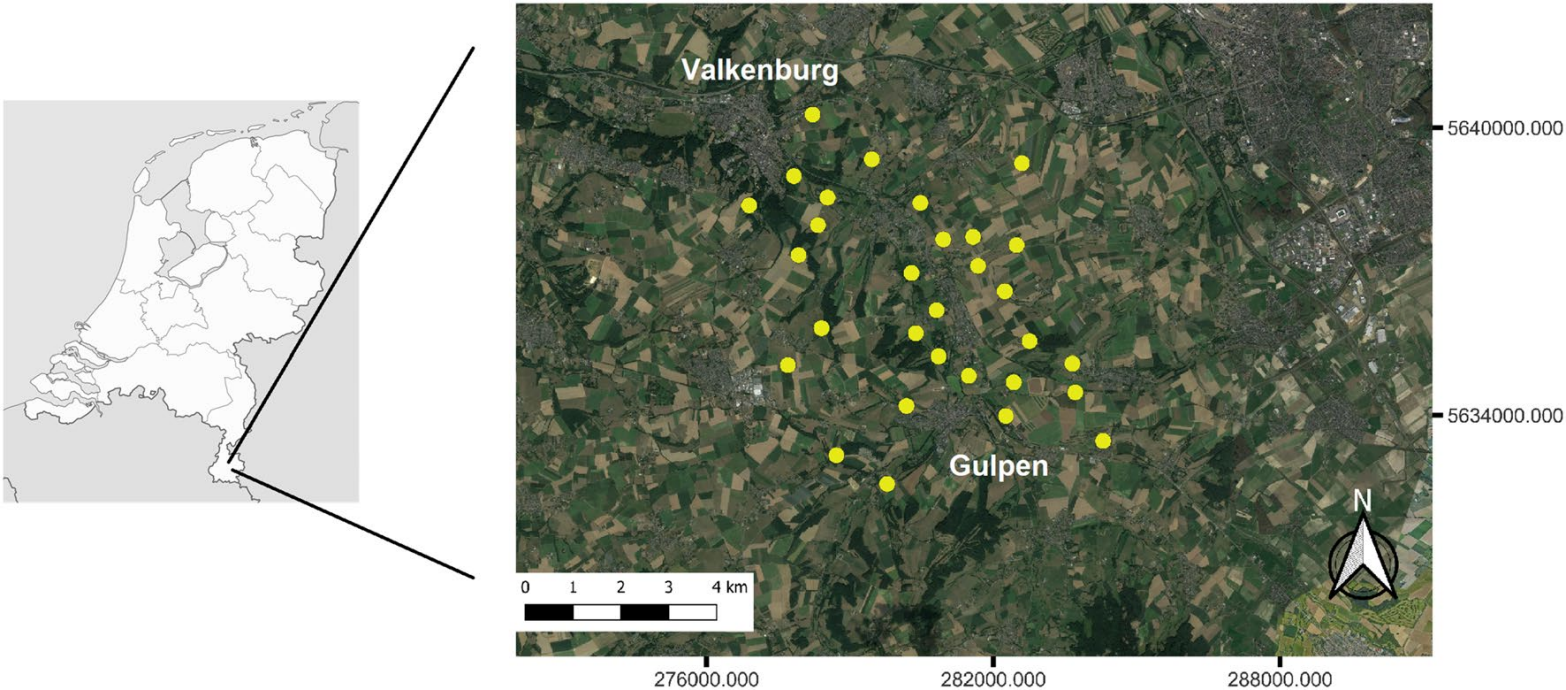
The study areas located in the Southeast of the Netherlands. The 30 transects within each study area are indicated by yellow dots (Basemap: Google Earth map as of August 2022).

The study areas (approximately 70 km$^2$ with elevations ranging from 70 to 171 m asl) are located in the southeast of the Netherlands, near the village of Gulpen (Fig. 2). Thirty grasslands representing a range of land use intensities, from nutrient-poor, biodiversity-rich semi-natural grasslands to intensively fertilized areas, were chosen in order to test the proposed approach. Management of the grasslands included mowing (16 sites), grazing (10 sites) and mixed regimes (4 sites), ranging in intensity from one to five uses per year (details in Appendix Table 1). Data collection for this study took place before the first cut but extensive grazing (<2 LSU/ha) had occurred at most grazed plots. Percent herb cover ranged from 0.1% to 69%, with the most dominant species in terms of flower cover being *Ranunculus repens*, *R. acris* and *R. bulbosus*, *Leucanthemum vulgare*, *Trifolium pratense*, *Bellis perennis* and *Taraxacum sp.* (all >5% of the total flower area over all transects). The study areas are part of the experimental biodiversity area network of the EU Showcase project https://showcase-project.eu/. By selecting semi-natural, extensively utilized, and intensely managed grasslands from diverse regions, we reduced spatial clustering of distinct grassland types.

### Field data

#### Collection

In each study area, a transect measuring 150 m by 1 m was established and divided into three equal sections of 50 m. These transects were visibly marked with Ground Control Points (GCP) plates that could be identified by UAV imagery. GCP were positioned from the edge to the center of the grassland, covering differences in elevation heterogeneity within the grassland helping successively to find our sampling locations on the images. To ensure a sampling of distinct bee populations, adjacent transects were generally separated by distances greater than 500 m^56^. Previous studies^56^ have shown that, although large-bodied bees like bumblebees can forage at distances of a few kilometers, their primary foraging distances are shorter, typically ranging between 250 m and 550 m. Smaller wild bees tend to forage even closer to their nests. Along each transect, surveys were conducted for both bees and flowers. Transect walks, a standard method for studying plant-pollinator associations, were used to count both wild bees and honeybee (*Apis mellifera*)^83^. The transects were surveyed by two observers who counted all bees within a meter in front of them while slowly walking along the transect for 15 min, excluding the time required for handling caught specimens. Species were identified using identification keys specific to Dutch Apidae^17,46,47^. While distinctive species could be identified in the field, other specimens were collected and identified in the laboratory using stereo-microscopes and, in some cases, expert consultation. Subsequent to the bee surveys, flower surveys were conducted in each transect at which the number of flowers within the 150 m x 1 m transect was counted per species^70^. Hence, only flowering species richness was recorded and abundance was measured in terms of flowering. Flower surveys were generally conducted on the same day as the bee surveys, but due to logistical constraints, some grasslands were surveyed one or two days before or after the bee surveys (details in Appendix Table 1). The surveys were conducted between May 12th and 31st, 2021, from 10 a.m. to 5 p.m., under favorable weather conditions, which included dry conditions, more than 50% sunlight, temperatures of at least 15 degrees Celsius, and wind speeds below 2 Beaufort.

#### Ground-based diversity indices

The ground-based flower diversity was calculated using the species richness, namely the number of different flower species per transect. Also for the characterization of bee diversity, we relied on species richness. Bee abundance was defined as the total number of bees counted along each transect.

### UAV Data Acquisition and Data Processing

The UAV data were acquired simultaneously with the field survey between May 12th and 31st, 2021. A RGB Zenmuse X5 camera (16.0 MP, 17.3 x 13.0 mm sensor) with an integrated RTK GPS was carried by the UAV “DJI Matrice 210 RTK”. To simplify the production of the final point cloud and the digital elevation model, the images were taken at an overlapping rate of 80%. All flights were conducted at a height of approximately 20 m above the ground. The average spatial resolution of the resulting UAV images is 0.5 cm.

The Agisoft Metashape Professional Edition software was used to analyze and process the UAV images following three main procedural stages: image alignment, dense point cloud creation, and inference of the digital elevation model. In the first step, set with “high” accuracy, the software extracted features within the images and matched them to produce a sparse 3D point cloud. At this stage, the software automatically detected the precise features of the GCP and extracted the GPS coordinates for each of them. We maintained the “high” accuracy setting during the construction of the dense point cloud, which was subsequently exported as a LAS file. The mean point density for all 30 areas was 700 points/m$^2$ while the vertical resolution was around 15 mm. The Digital Surface Model (DSM) was derived at different spatial resolutions (10 cm, 25 cm, and 50 cm) using the “dsmtin” algorithm of the “rasterize_canopy” function of the R package “lidR”^68^. This algorithm uses the Delaunay triangulation method to connect the points in the point cloud, forming a network of non-overlapping triangles. The resulting triangular irregular network (TIN) represents the surface, and rasterization is then applied to convert this TIN into a gridded DSM, providing a comprehensive representation of the terrain and vegetation structure. The Digital Terrain Model (DTM) was derived using the same function but with a prior filtering of the point cloud, selecting the lowest points every 50 cm. Finally, the CHM was derived by taking the difference between the DSM and DTM. The decision to set the finest spatial resolution at 10 cm was primarily driven by computational considerations.

### Heterogeneity index

HH was calculated using the CHM at different spatial resolutions (10 cm, 25 cm, and 50 cm) with four different heterogeneity indices: Rao’s Q index, the CV, the Berger–Parker index, and the Simpson’s D index^64^.

The Rao’s Q index, originally developed by Rao^55^, was later recommended by Botta–Dukát^5^ as a functional diversity index in ecology. Subsequently, Rocchini et al.^62^ introduced this measure as a heterogeneity index for remote sensing data, employing the following Eq. (1):

1 $$\begin{aligned} Q=\sum _{i,j=1}^{N}{d_{ij}\times p_i \times p_j} \end{aligned}$$

where:

Q = Rao’s Q index, used in remote sensing application

$p_i$ = $p_j$ = 1/*N* = relative abundance of pixel i, j in a selected area (i.e. in our case, raster over the transects) composed of N pixels

$d_{ij}$ = distance/dissimilarity between pixel i and j ($d_{ij}$ = $d_{ji}$ and $d_{ii}=0$)

We determined $d_{ij}$ as the Euclidean distance using a solitary layer (CHM raster).

The CV, widely employed as a measure of heterogeneity in various ecological studies^22,35^, is calculated using the following Eq. (2):

2 $$\begin{aligned} CV = (SD/\overline{x}) \times 100 \end{aligned}$$

where:

CV= Coefficient of Variation

SD= Standard Deviation of the pixel values within a selected area

$\overline{x}$ = mean of the pixel values within a selected area

The Berger–Parker index is often used as a heterogeneity index in ecological studies and also with remote sensing data, it provides a measure of species/pixel dominance within a given community/data-set^86^. It has been calculated using the following Eq. (3):

3 $$\begin{aligned} BP = \frac{n_{\text {max}}}{N} \end{aligned}$$

where:

BP is the Berger–Parker heterogeneity index

- $n_{\text {max}}$ is the abundance of the most dominant pixel value in the data-set

- N is the total abundance of all pixels in the data-set.

The Simpson’s D index is a diversity assessment measure frequently employed in ecology^13,33^. It can also serve as a heterogeneity measure with remote sensing data, relying solely on the relative abundance of pixels within the specific area^64^. It is calculated as (Eq. 4):

4 $$\begin{aligned} \textit{D}=\sum _{i=1}^{n} p_i^2 \end{aligned}$$

where: *D* = Simpson index. *n*= total number of pixel’s value. $p_i$ = relative abundance of a pixel value in a CHM raster plot.

### Validation of the UAV DTM and CHM

DSM and DTM with a spatial resolution of 50 cm derived from local Li-DAR data collected as part of an national ALS LiDAR campaign carried out between 2020 and 2022 (AHN4 data-set, freely available for download here: https://geotiles.nl/) were used to validate the UAV digital models. AHN datasets are systematically gathered every few years for all of the Netherlands, by multiple operators and sensors, where the exact specifications may vary over time and space. AHN4 pointclouds have a vertical resolution of 13 mm and a density of 10-14 point/m2. In our study area, the LiDAR flight for AHN4 was conducted on February 18th, 2021. During this season, the grassland vegetation is very low, resulting in the DSM and DTM having equal elevations, effectively yielding a CHM value of zero. For this reason, we decided to validate the UAV-DTM with the LiDAR-DTM using 10 random points within each study area (300 points in total). Additionally, we validated the CHM over multi-annual visible vegetation-patch (e.g., small shrubs) that could be visible in both the UAV-CHM and LiDAR-CHM. We randomly selected a point over each multi-annual visible vegetation for each study area (29 points in total) and correlated the digital models using linear regression.

For both the DTM and CHM, the coefficient of determination ($R^2$) was used to estimate the goodness of fit of the model, while the *P value* was used to measure its statistical significance.

### Workflow

**Figure 3:**
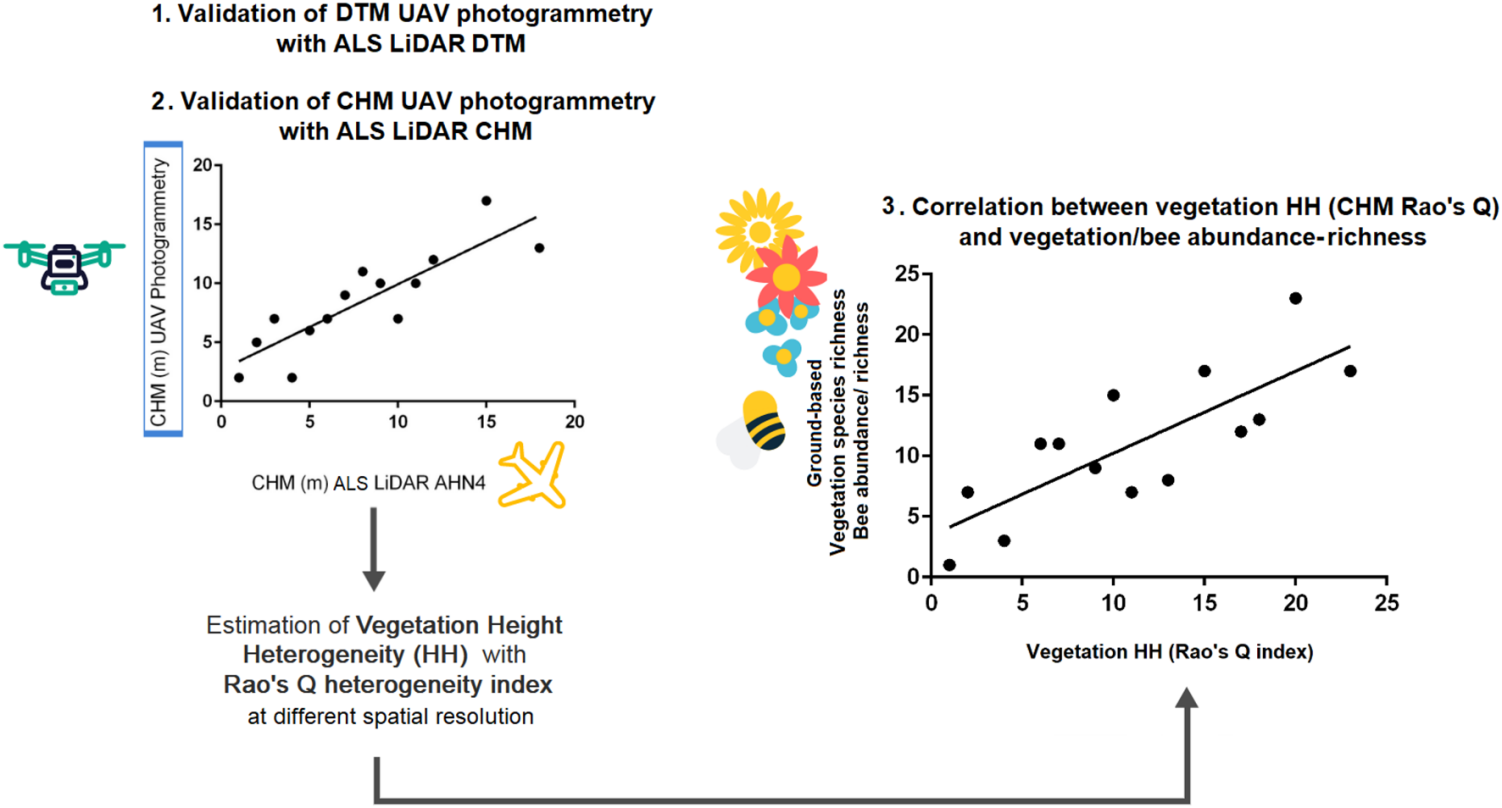
The image shows the workflow of the proposed approach.

The approach proposed in this study is summarized in Fig. 3. Firstly (point 1), we validated the UAV DTM and CHM with DTM and CHM derived from the local ALS data. Then (point 2), for each transect, we estimated HHs using the UAV CHM data at different spatial resolutions (10 cm, 25 cm, and 50 cm) with four different heterogeneity indices (Rao’s Q index, CV, Berger–Parker index and Simpson’s D index). Subsequently, we performed linear regression analyses to correlate the HHs with the ground-based flower and bee diversity and bee abundance. The coefficient of determination ($R^2$) was used to estimate the goodness of fit of the model, while the *P value* was used to measure its statistical significance.

## Results

**Figure 4:**
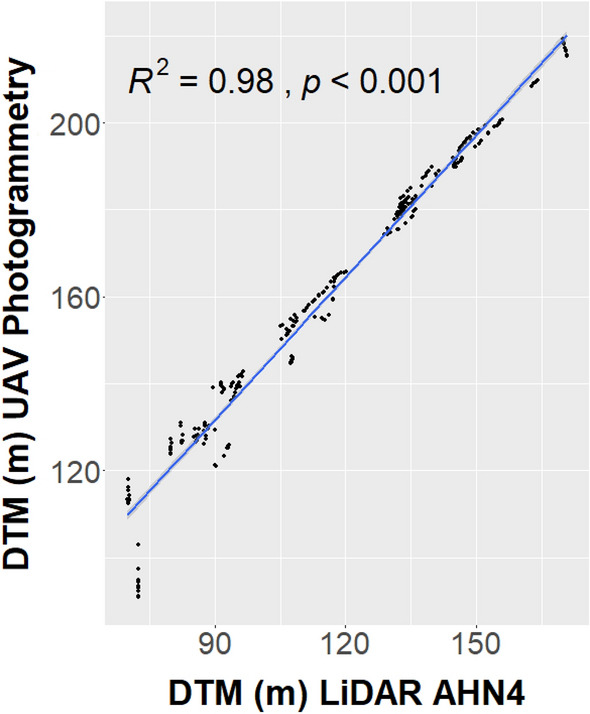
The validation of the DTM derived from UAV photogrammetry with the local LiDAR DTM AHN4 is shown with the blue line.

The validation of the DTM derived from UAV photogrammetry with local ALS DTM LiDAR data (AHN4 data-set) at a spatial resolution of 50 cm is shown in Fig. 4. The linear regression analysis yielded a positive relationship and strong correlation between the two variables. The correlation between the two variables is significant (p-value < 0.05), with a goodness of fit of 0.98. The UAV-derived DTM tends to be higher than the LiDAR DTM with a systematic average offset of 44 m (calculated as the difference of the mean’s datasets).

**Figure 5:**
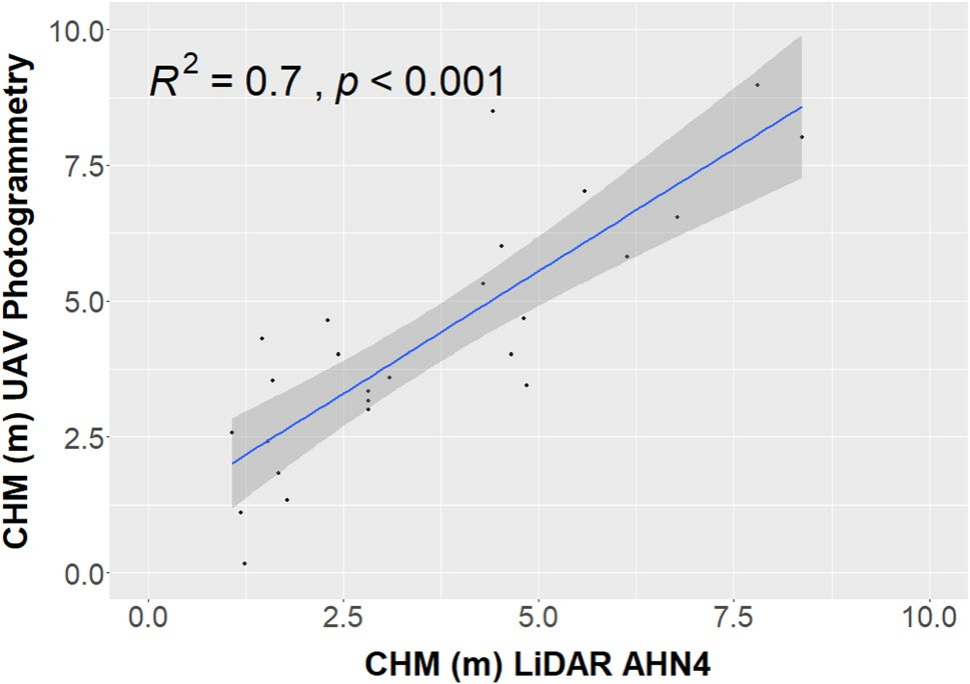
Validation of the CHM derived from UAV photogrammetry with the local LiDAR CHM AHN4.

The validation of the CHM derived from UAV photogrammetry with local ALS CHM LiDAR data (AHN4 data-set) at a spatial resolution of 50 cm is shown in Fig. 5. Similar to the DTM, the linear regression analysis shows a positive relationship, and the UAV CHM tends to overestimate the LiDAR CHM with an offset of 1002 m. This offset may be attributed to various factors, including seasonality differences (LiDAR data were collected in February during the leaf-off season, while photogrammetric data were acquired in early spring in May), data processing (methodological distinction arises from the inability to directly calculate the DTM with photogrammetry that was derived from the DSM) and differences in the used processing algorithms employed for DTM and DSM assessment. Despite the presence of this offset, the correlation between the two variables remains statistically significant (p-value < 0.05), and the linear model exhibits a commendable goodness of fit at 0.71.

**Figure 6:**
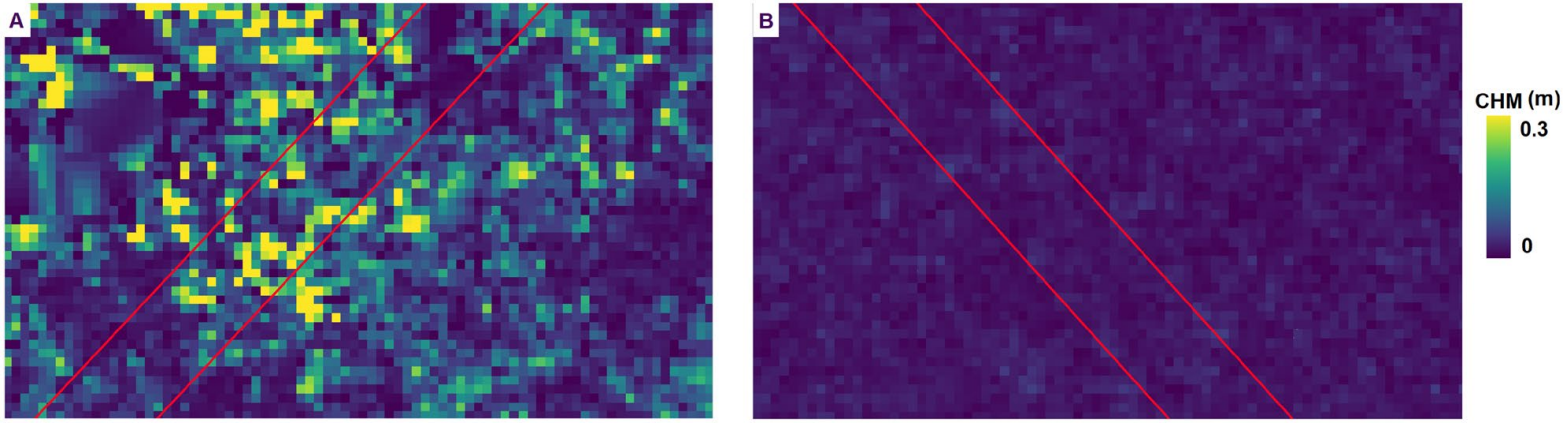
Two transects (in red) characterized by different height heterogeneity. Sub-figure A shows a CHM of a transect characterized by high height heterogeneity (heterogeneous CHM ranging from 0 to 0.3 m), while sub-figure B shows a transect with low height heterogeneity (homogeneous CHM with values ranging from 0 m to 0.1 m).

Figure 6 shows a study area with two different vegetation structure. In the middle of the figure is shown a stripe of grass characterized by a higher vegetation structure complexity and high HH while on the side grassland with low HH. Sub-figure A shows the RGB image, sub-figure B the CHM derived from the photogrammetric point cloud showed in sub-figure C.

**Figure 7:**
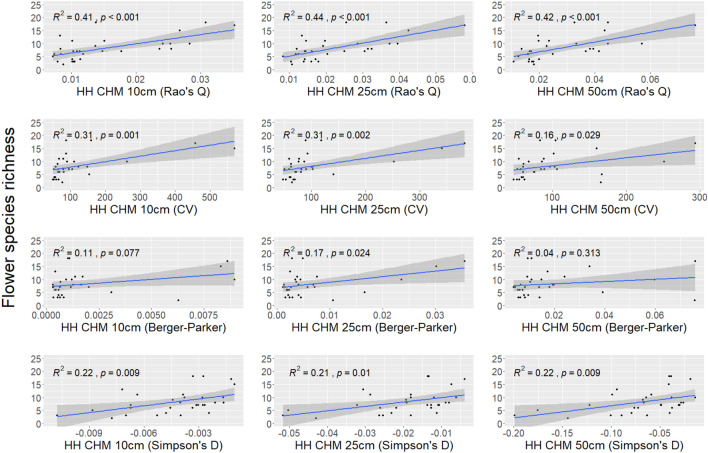
Correlation between the ground-based flower diversity and the HH calculated with the four heterogeneity indices (Rao’s Q, CV, Berger–Parker and Simpson’s D) derived from UAV CHM at 10 cm, 25 cm and 50 cm.

The correlation between the flower diversity and calculated HH with different heterogeneity indices (Rao’s Q index, CV, Berger–Parker, and Simpson’s D) using the CHM at 10 cm, 25 cm, and 50 cm derived from UAV photogrammetry is shown in Fig. 7. All the correlations are positive and significant, except when the HH was calculated with the Berger–Parker index using a CHM of 10 cm and 50 cm. The highest $R^2$ values were obtain when the HH was calculated with the Rao’s Q index. In this case, the coefficient of determination range between 0.41 (UAV CHM spatial resolution of 10 cm) and 0.44 (UAV CHM spatial resolution of 25 cm).

**Figure 8:**
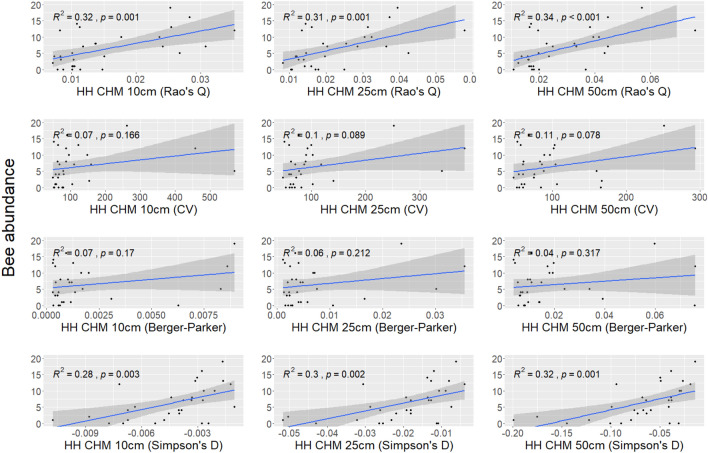
Correlation between ground-based bee abundance and HH calculated with the four heterogeneity indices (Rao’s Q, CV, Berger–Parker, and Simpson’s D) derived from UAV CHM at 10 cm, 25 cm, and 50 cm.

Figure 8 shows the correlation between the bee abundance and the HH calculated with different heterogeneity indices (Rao’s Q index, CV, Berger–Parker, and Simpson’s D) using the CHM at 10 cm, 25 cm, and 50 cm derived from UAV photogrammetry. In this case, the correlations are all positive, and significant only when the HH was calculated with the Rao’s Q and Simpson’s D indices. Generally, the $R^2$ values are lower than the ones derived from the correlation between HH and flower diversity. Higher $R^2$ are associated with HH calculated using the Rao’s Q index. The coefficient of determination ranges between 0.31 (UAV CHM spatial resolution of 25 cm) and 0.34 (UAV CHM spatial resolution of 50 cm).

**Figure 9:**
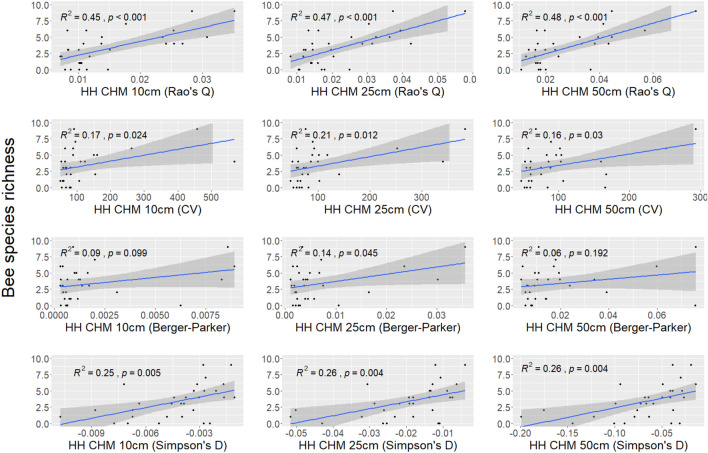
Correlation between ground-based bee diversity and HH calculated with the four heterogeneity indices (Rao’s Q, CV, Berger–Parker, and Simpson’s D) derived from UAV CHM at 10 cm, 25 cm, and 50 cm.

Finally, the correlation between bee diversity and HH calculated with different heterogeneity indices (Rao’s Q index, CV, Berger–Parker, and Simpson’s D) using the CHM at 10 cm, 25 cm, and 50 cm derived from UAV photogrammetry is shown in Fig. 9. Also in this case, positive correlations persist, with the Rao’s Q index yielding the highest $R^2$ values, while the Simpson’s D index shows a comparatively modest correlation with HH. They are significant, except when the HH was calculated with the Berger–Parker index (with CHM at 10 cm and 50 cm).

## Discussion

This paper introduces a new approach to estimate flower diversity, which can be used as an indicator of bee abundance and diversity in grassland ecosystems. Our study builds upon previous studies^77^ that identified UAV images, analyzed through various machine learning algorithms, as a reliable proxy for bee diversity and abundance. However, with this innovative HVH approach, we delve deeper into unraveling the intricate relationship between grassland structural heterogeneity and its impact on bee diversity. The method utilizes UAV RGB images to create a 3D model of the vegetation structure through photogrammetric analysis. By applying different heterogeneity indices, we derived information on vegetation HH, which showed a positive correlation with ground-based measures of flower diversity, bee diversity, and bee abundance. These findings serve as a proof of concept, demonstrating the potential of UAV imagery to accurately evaluate the habitat structure as a crucial element of grassland habitat quality for bees. The findings of this study provide valuable insights into the use of UAV imagery and HH in estimating biodiversity at a local scale, specifically in grassland ecosystems. The results indicate that vegetation height heterogeneity, as measured through UAV-derived CHMs, can serve as a proxy for flower diversity and, consequently, bee diversity and abundance.

### Height Variation Hypothesis in grassland ecosystem: Advantages, Contrasts, and Ecological Implications

The proposed approach relies on the theory behind the HVH which, unlike its counterpart (the Spectral Variation Hypothesis -SVH-), offers several significant advantages. Being based on vertical structural heterogeneity, the HVH is not susceptible to certain factors such as the spectral resolution of the optical images^45,60^, by noise introduced by the soil properties which can negatively affect the accuracy of biodiversity assessments^22^ and by the atmospheric conditions such as haze, aerosols, and cloud cover^61^.

This study provides a novel application of the HVH with UAV images in grasslands. The results indicate the potential of photogrammetric analysis for biodiversity assessment in grasslands, contributing to the understanding of vegetation structure and its relationship with biodiversity. As shown in other studies^50,85^, the high-resolution cameras mounted to UAVs allow capturing of detailed images, enabling the assessment of fine-scale heterogeneity of intensively and extensively managed grasslands. The proposed approach highlights the capability of UAVs to assess grassland vegetation structure and heterogeneity, providing detailed information about the vertical complexity and variability of the vegetation, critical information for understanding ecosystem dynamics, biodiversity, and habitat suitability for various organisms^50^.

Other approaches have been developed to assess these aspects by using UAV data; recent studies for example focused on the evaluation of flower abundance as a proxy for diversity and abundance of bees^11,77^. These approaches often rely on machine learning algorithms, which necessitate meticulously curated and representative training data-sets that, due to their time-consuming and resource-intensive nature, can potentially hinder scalability and applicability in certain contexts^8^. Moreover, the representativeness of the training data-set is critical to ensure the generalizability of the algorithm’s performance. These challenges can impede the scalability and applicability of machine learning-based approaches under conditions, where there are no comprehensive and diverse training data-sets^74^. Furthermore, machine learning algorithms may exhibit limitations in their ability to capture the full complexity of ecological dynamics. They rely on patterns and associations learned from the training data-sets, which may not encompass the entirety of the intricate relationships within an ecosystem. Consequently, the predictive power of machine learning models may be limited when confronted with novel or complex ecological scenarios that deviate from the patterns represented in the training data-set^48^.

The findings obtained by our analytical approach hold significant relevance for ecological studies for multiple reasons. Understanding the vertical complexity and variability of grassland vegetation provides insights also into habitat heterogeneity and resource availability for various organisms, including plants, insects, birds, and small mammals^3,24,49^. Different species have specific habitat preferences and requirements based on their vertical distribution within the grassland. Assessing grassland structure helps to understand the composition, distribution, and abundance of species within the ecosystem^52^. It would be intriguing to explore whether there exists a correlation between grassland structure and the various ecosystem processes and services such as nutrient cycling, carbon storage, water infiltration, and energy fluxes. If such a correlation is found, our approach could be utilized to achieve more precise mapping of these significant ecosystem services, surpassing the current methods employed. Additionally, the information on grassland structure can be integrated with other environmental data, such as soil properties and landscape features, to gain a more holistic understanding of the ecological dynamics and drivers in grassland ecosystems^67^. Furthermore, the proposed approach could be used to assess changes in grassland structure as a results of land management practices, ecological succession, and of the impacts of disturbances such as grazing, fire, or land-use changes. Monitoring and understanding these structural changes are essential for effective conservation and management of grassland ecosystems^15,25,32^.

### UAVs in Bee Habitat Monitoring: Challenges and Prospects

UAV-based methods have emerged as promising tools for monitoring habitat quality for bee pollinator communities, primarily due to their affordability^23^. These methods allow different operators, including researchers, farmers, and ecologists, to acquire high spatial resolution data from various sensors simultaneously, covering extensive areas within a short time of data collection. Furthermore, the “on-demand” approach facilitated by UAVs enables capturing specific stages of vegetation phenology, such as flowering time, particularly in regions characterized by high cloud cover^12,44^. These capabilities provide valuable insights into the temporal dynamics of plant-pollinator interactions. However, despite their potential, several challenges must be addressed before UAV-based methods can be routinely deployed at large spatial scales. Challenges arising may involve issues related to data processing, sensor calibration, image analysis algorithms, and the development of standardized protocols to ensure data comparability and reliability. Addressing these challenges paves the way for a visionary application, where UAVs, equipped with advanced sensors, facilitate large-scale macroecological studies. This approach enables real-time data acquisition, enhancing our understanding of spatial patterns, biodiversity dynamics, and ecosystem processes across diverse landscapes.

The impact of the spatial resolution of UAV data on the correlation between grassland structural metrics (such as HH) and flower and bee diversity, as well as bee abundance was investigated in this study. Based on our results, the spatial resolution of UAV data does not play a critical role in the correlations between vegetation assessment variables (such as flower diversity, bee abundance, and bee diversity) and HH calculated using different heterogeneity indices. The correlations between these variables remain positive and significant across different spatial resolutions (10 cm, 25 cm, and 50 cm) derived from UAV photogrammetry. This finding aligns with the results reported in several other studies examining the influence of spatial resolution on vegetation assessment using UAV imagery. For instance,^37^ demonstrated that species classification in a heterogeneous grassland using high spatial resolution UAV imagery was not significantly affected by spatial resolution. Similarly, the impact of spatial resolution on the classification of vegetation types in highly fragmented planting areas based on UAV hyperspectral images was found to be limited^36^. Different studies^11,29^ highlighted that the use of micro-UAV with relatively low spatial resolution still provide valuable information for assessing vegetation structure and for long-term monitoring purposes. On the other hand, it is important to note that the relationship between the high spatial resolution of optical remote sensing data and its correlation with ground-based ecological data is a complex matter^42^. Different studies^60,66,77^ have shown that higher spatial resolution can lead to higher correlations with ground-based data. It is recognized that images with coarse spatial resolution may integrate the spectral signature of various vegetation elements, making it challenging to identify boundaries between spatial entities and potentially resulting in mixed signals at the pixel scale^19,45^. These results imply that drone flights can also be conducted at higher altitudes and thus cover larger areas in a single flight (at a lower spatial resolution), enabling more efficient data collection.

### Insights from Heterogeneity Indices

Regarding the evaluation of the use of different heterogeneity indices, our results demonstrated the usefulness of the Rao’s Q index in assessing the vegetation HH across areas of intensive and extensive grassland management. This heterogeneity index, widely used as a spectral heterogeneity index in studies on SVH^39,51,62,75^ offers the advantage of coupling both the relative abundance and the pixel values (as quantified by the Euclidean distance between the pixel values)^78^, thus capturing the complete structural information derived from the heterogeneity of the photogrammetric outcomes. This index, when applied with a single layer or raster as in our study, can effectively serve as a proxy for heterogeneity by narrowing it down to variance using half of the squared Euclidean distance (1/2 $d_{ij}^2$) (for further details on the mathematical characteristics of Rao’s Q, we refer to^57–59,62^). On the other hand, other indices evaluated in our work, proved rather inefficient in assessing HH: the CV rely only on the distance between the pixel values while the Simpson’s D and the Berger–Parker index rely solely on the relative abundance of CHM pixels within a specific raster or an area of interest^62^; given the exceptional precision of our photogrammetric point cloud, approximately 15 mm, the likelihood of distinct pixels sharing identical values is for this reason significantly minimized. Consequently, they fail to adequately characterize the entire heterogeneity of vegetation heights, which depend on both the actual values of vegetation height and their distribution and relative frequency. One concern in this study revolves around the utilization of the CHM as the sole metric for assessing the HH, without considering other metrics or additional digital layers, such as optical data that might be related to vegetation structure. The decision to focus solely on the CHM had two main reasons. Firstly, the primary objective of this study was to investigate the feasibility of utilizing RGB UAV images to assess vegetation structure complexity for estimating HH and flower and bee diversity and bee abundance. Secondly, choice was guided by the findings of Tamburlin et al.^72^, who, testing the HVH with LiDAR data, evaluated various LiDAR metrics (such as entropy and standard deviation of point cloud distribution, percentage of returns above mean height) for HH estimation and demonstrated that the CHM was the most effective metric to characterize vegetation HH.

Another concern that could arise is related to the accuracy of the UAV derived CHM. While the CHMs derived from UAV photogrammetry showed a robust correlation with local CHM LiDAR data, there may still be some differences in accuracy and precision. We acknowledge that photogrammetry techniques may not capture true ground points accurately, especially in areas with dense and short grass. One possible way to enhance the precision of our approach could be the utilization of LiDAR technology mounted on UAVs that can provide more precise and detailed measurements of vegetation structure and topography^4^, offering valuable information on floral resources and bee foraging habitats. However, it is worth noting that LiDAR-equipped UAVs are currently considered expensive, which can limit their widespread use. Furthermore recent studies indicate that these systems may not necessarily exhibit significantly improved performance in acquiring accurate DSMs within closed vegetation canopies^73^. It is important to clarify that our primary interest lies in assessing the vertical variation within the point cloud rather than obtaining absolute values for ground surface measurements. To address this concern, we employed a methodology focused on analyzing the amount of variation in vertical points rather than relying on precise ground measurements, allowing to evaluate the relative differences in elevation values between different areas, which can still provide valuable insights into the landscape dynamics and terrain characteristics.

## Conclusions

This study demonstrates the potential of UAV imagery and the HVH concept for estimating biodiversity at a local scale in grassland ecosystems. The results suggest that vegetation HH, as assessed through UAV-derived CHMs, can serve as a reliable proxy for flower diversity, bee diversity, and abundance. The use of UAVs, with the ability to assess species diversity and provide information on grassland structure, offers a cost-effective and standardized approach to monitor and manage grassland ecosystems, providing valuable information for conservation efforts and advancing ecological research. While our study serves as an initial application, further analysis in diverse grassland areas using various heterogeneity indices is necessary to establish the generalizability of the approach. Additionally, this approach could be extended to assess biodiversity not only of bees but also of other insects such as spiders or butterflies. Further analysis could focus on integrating optical information, such as flower cover estimation, or spectral variability data, with structural information from UAVs enhancing the depth of biodiversity characterization. We propose that ecologists, botanists, and farmers can employ our approach, utilizing UAV images and photogrammetric analysis in order to assess habitat heterogeneity, as a preliminary analysis for the estimation of bee diversity and abundance.

### Supplementary Information

Supplementary Information.

## Data Availability

The datasets used and/or analysed during the current study available from the corresponding author on reasonable request.

## References

[1] Akinbiola S, Salami AT, Awotoye OO, Popoola SO, Olusola JA (2023). Application of UAV photogrammetry for the assessment of forest structure and species network in the tropical forests of southern nigeria. Geocarto Int..

[2] Anderle M, Brambilla M, Hilpold A, Matabishi JG, Paniccia C, Rocchini D, Rossin J, Tasser E, Torresani M, Tappeiner U (2023). Habitat heterogeneity promotes bird diversity in agricultural landscapes: Insights from remote sensing data. Basic Appl. Ecol..

[3] Banaszak, J. Effect of habitat heterogeneity on the diversity and density of pollinating insects. *Interchanges of insects between agricultural and surrounding landscapes* (2000), 123–140.

[4] Bartholomeus H, Calders K, Whiteside T, Terryn L, Krishna Moorthy SM, Levick SR, Bartolo R, Verbeeck H (2022). Evaluating data inter-operability of multiple UAV-lidar systems for measuring the 3d structure of savanna woodland. Remote Sens..

[5] Botta-Dukát Z (2005). Rao’s quadratic entropy as a measure of functional diversity based on multiple traits. J. Veg. Sci..

[6] Breeze TD, Bailey AP, Balcombe KG, Potts SG (2011). Pollination services in the UK: How important are honeybees?. Agricult. Ecosyst. Environ..

[7] Cavender-Bares J, Schneider FD, Santos MJ, Armstrong A, Carnaval A, Dahlin KM, Fatoyinbo L, Hurtt GC, Schimel D, Townsend PA (2022). Integrating remote sensing with ecology and evolution to advance biodiversity conservation. Nat. Ecol. Evolut..

[8] Christin S, Hervet É, Lecomte N (2019). Applications for deep learning in ecology. Methods Ecol. Evol..

[9] Curcio AC, Barbero L, Peralta G (2023). UAV-hyperspectral imaging to estimate species distribution in salt marshes: A case study in the Cadiz Bay (SW Spain). Remote Sens..

[10] da Silva SDP, Eugenio FC, Fantinel RA, de Paula Amaral L, dos Santos AR, Mallmann CL, dos Santos FD, Pereira RS, Ruoso R (2023). Modeling and detection of invasive trees using UAV image and machine learning in a subtropical forest in Brazil. Eco. Inform..

[11] de Castro AI, Shi Y, Maja JM, Peña JM (2021). UAVs for vegetation monitoring: Overview and recent scientific contributions. Remote Sens..

[12] De Sa NC, Castro P, Carvalho S, Marchante E, López-Núñez FA, Marchante H (2018). Mapping the flowering of an invasive plant using unmanned aerial vehicles: Is there potential for biocontrol monitoring?. Front. Plant Sci..

[13] DeJong, T. M. A comparison of three diversity indices based on their components of richness and evenness. *Oikos* (1975), 222–227.

[14] Dubayah R, Armston J, Healey SP, Bruening JM, Patterson PL, Kellner JR, Duncanson L, Saarela S, Ståhl G, Yang Z (2022). Gedi launches a new era of biomass inference from space. Environ. Res. Lett..

[15] Duelli P (1997). Biodiversity evaluation in agricultural landscapes: An approach at two different scales. Agricult. Ecosyst. Environ..

[16] Duncanson L, Kellner JR, Armston J, Dubayah R, Minor DM, Hancock S, Healey SP, Patterson PL, Saarela S, Marselis S (2022). Aboveground biomass density models for NASA’s global ecosystem dynamics investigation (GEDI) lidar mission. Remote Sens. Environ..

[17] Falk, S., and Lewington, R. *Veldgids bijen voor Nederland en Vlaanderen*. 2017.

[18] Feilhauer H, Doktor D, Schmidtlein S, Skidmore AK (2016). Mapping pollination types with remote sensing. J. Veg. Sci..

[19] Feilhauer H, Zlinszky A, Kania A, Foody GM, Doktor D, Lausch A, Schmidtlein S (2021). Let your maps be fuzzy!-class probabilities and floristic gradients as alternatives to crisp mapping for remote sensing of vegetation. Remote Sens. Ecol. Conserv..

[20] Gallai N, Salles J-M, Settele J, Vaissière BE (2009). Economic valuation of the vulnerability of world agriculture confronted with pollinator decline. Ecol. Econ..

[21] Gallmann, J., Schüpbach, B., Jacot, K., Albrecht, M., Winizki, J., Kirchgessner, N., and Aasen, H. Flower mapping in grasslands with drones and deep learning. *Front. Plant Sci. 12* (2021). 10.3389/fpls.2021.774965 PMC8864122 35222449

[22] Gholizadeh H, Gamon JA, Zygielbaum AI, Wang R, Schweiger AK, Cavender-Bares J (2018). Remote sensing of biodiversity: Soil correction and data dimension reduction methods improve assessment of *α*-diversity (species richness) in prairie ecosystems. Remote Sens. Environ..

[23] Gonzales, D., Hempel de Ibarra, N., and Anderson, K. Remote sensing of floral resources for pollinators–new horizons from satellites to drones. *Front. Ecol. Evolut. 10* (2022).

[24] Hovick TJ, Elmore RD, Fuhlendorf SD (2014). Structural heterogeneity increases diversity of non-breeding grassland birds. Ecosphere.

[25] Howison RA, Piersma T, Kentie R, Hooijmeijer JC, Olff H (2018). Quantifying landscape-level land-use intensity patterns through radar-based remote sensing. J. Appl. Ecol..

[26] Hui G, Zhang G, Zhao Z, Yang A (2019). Methods of forest structure research: A review. Curr. For. Rep..

[27] Kleijn D, Kohler F, Báldi A, Batáry P, Concepción E, Clough Y, Díaz M, Gabriel D, Holzschuh A, Knop E (2009). On the relationship between farmland biodiversity and land-use intensity in Europe. Proc. Royal Soc. B Biol. Sci..

[28] Kleijn D, Winfree R, Bartomeus I, Carvalheiro LG, Henry M, Isaacs R, Klein A-M, Kremen C, M’gonigle LK, Rader R (2015). Delivery of crop pollination services is an insufficient argument for wild pollinator conservation. Nat. Commun..

[29] Kolarik NE, Gaughan AE, Stevens FR, Pricope NG, Woodward K, Cassidy L, Salerno J, Hartter J (2020). A multi-plot assessment of vegetation structure using a micro-unmanned aerial system (UAS) in a semi-arid savanna environment. ISPRS J. Photogramm. Remote. Sens..

[30] Kremen, C., Chaplin-Kramer, R., et al. Insects as providers of ecosystem services: crop pollination and pest control. In *Insect conservation biology: proceedings of the royal entomological society’s 23rd symposium* (2007), CABI Publishing Wallingford, UK, 349–382.

[31] Kremen C, Williams NM, Thorp RW (2002). Crop pollination from native bees at risk from agricultural intensification. Proc. Natl. Acad. Sci..

[32] Kuemmerle T, Erb K, Meyfroidt P, Müller D, Verburg PH, Estel S, Haberl H, Hostert P, Jepsen MR, Kastner T (2013). Challenges and opportunities in mapping land use intensity globally. Curr. Opin. Environ. Sustain..

[33] Kumar P, Dobriyal M, Kale A, Pandey A, Tomar R, Thounaojam E (2022). Calculating forest species diversity with information-theory based indices using sentinel-2a sensor’s of Mahavir Swami wildlife sanctuary. PLoS ONE.

[34] Lang, N., Jetz, W., Schindler, K., and Wegner, J. D. A high-resolution canopy height model of the earth. arXiv preprint arXiv:2204.08322 (2022). 10.1038/s41559-023-02206-6 PMC10627820 37770546

[35] Levin N, Shmida A, Levanoni O, Tamari H, Kark S (2007). Predicting mountain plant richness and rarity from space using satellite-derived vegetation indices. Divers. Distrib..

[36] Liu M, Yu T, Gu X, Sun Z, Yang J, Zhang Z, Mi X, Cao W, Li J (2020). The impact of spatial resolution on the classification of vegetation types in highly fragmented planting areas based on unmanned aerial vehicle hyperspectral images. Remote Sens..

[37] Lu B, He Y (2018). Optimal spatial resolution of unmanned aerial vehicle (UAV)-acquired imagery for species classification in a heterogeneous grassland ecosystem. GIScience Remote Sens..

[38] Melville B, Lucieer A, Aryal J (2019). Classification of lowland native grassland communities using hyperspectral unmanned aircraft system (UAS) imagery in the tasmanian midlands. Drones.

[39] Michele, T., Duccio, R., Marc, Z., Ruth, S., and Giustino, T. Testing the spectral variation hypothesis by using the rao-q index to estimate forest biodiversity: Effect of spatial resolution. In *IGARSS 2018-2018 IEEE International Geoscience and Remote Sensing Symposium* (2018), IEEE, 1183–1186.

[40] Moeslund JE, Zlinszky A, Ejrnæs R, Brunbjerg AK, Bøcher PK, Svenning J-C, Normand S (2019). Light detection and ranging explains diversity of plants, fungi, lichens, and bryophytes across multiple habitats and large geographic extent. Ecol. Appl..

[41] Moudrỳ V, Cord AF, Gábor L, Laurin GV, Barták V, Gdulová K, Malavasi M, Rocchini D, Stereńczak K, Prošek J (2023). Vegetation structure derived from airborne laser scanning to assess species distribution and habitat suitability: The way forward. Divers. Distrib..

[42] Moudrỳ V, Keil P, Gábor L, Lecours V, Zarzo-Arias A, Barták V, Malavasi M, Rocchini D, Torresani M, Gdulová K (2023). Scale mismatches between predictor and response variables in species distribution modelling: A review of practices for appropriate grain selection. Prog. Phys. Geogr. Earth Environ..

[43] Moudrỳ V, Moudrá L, Barták V, Bejček V, Gdulová K, Hendrychová M, Moravec D, Musil P, Rocchini D, Št’astnỳ K (2021). The role of the vegetation structure, primary productivity and senescence derived from airborne lidar and hyperspectral data for birds diversity and rarity on a restored site. Landsc. Urban Plan..

[44] Müllerová J, Brna J, Bartaloš T, Dvořák P, Vítková M, Pyšek P (2017). Timing is important: Unmanned aircraft vs. satellite imagery in plant invasion monitoring. Front. Plant Sci..

[45] Nagendra H, Rocchini D (2008). High resolution satellite imagery for tropical biodiversity studies: The devil is in the detail. Biodivers. Conserv..

[46] Nieuwenhuijsen, H., & Peeters, T. *Nederlandse bijen op naam brengen. Deel 1. - Stichting Jeugdbondsuitgeverij, ’s Graveland* (2015).

[47] Nieuwenhuijsen, H., Peeters, T., & Dijkshoorn, D. *Nederlandse bijen op naam brengen. Deel 2. - Stichting Jeugdbondsuitgeverij, ’s Graveland*. (2020).

[48] Olden JD, Lawler JJ, Poff NL (2008). Machine learning methods without tears: A primer for ecologists. Q. Rev. Biol..

[49] Palmeirim AF, Figueiredo MS, Grelle CEV, Carbone C, Vieira MV (2019). When does habitat fragmentation matter? A biome-wide analysis of small mammals in the Atlantic forest. J. Biogeogr..

[50] Peciña MV, Bergamo TF, Ward R, Joyce C, Sepp K (2021). A novel UAV-based approach for biomass prediction and grassland structure assessment in coastal meadows. Ecol. Ind..

[51] Perrone M, Di Febbraro M, Conti L, Divíšek J, Chytrỳ M, Keil P, Carranza ML, Rocchini D, Torresani M, Moudrỳ V (2023). The relationship between spectral and plant diversity: Disentangling the influence of metrics and habitat types at the landscape scale. Remote Sens. Environ..

[52] Petermann JS, Buzhdygan OY (2021). Grassland biodiversity. Curr. Biol..

[53] Potapov P, Li X, Hernandez-Serna A, Tyukavina A, Hansen MC, Kommareddy A, Pickens A, Turubanova S, Tang H, Silva CE (2021). Mapping global forest canopy height through integration of gedi and landsat data. Remote Sens. Environ..

[54] Potts, S. G., Ngo, H. T., Biesmeijer, J. C., Breeze, T. D., Dicks, L. V., Garibaldi, L. A., Hill, R., Settele, J., & Vanbergen, A. The assessment report of the intergovernmental science-policy platform on biodiversity and ecosystem services on pollinators, pollination and food production.

[55] Rao CR (1982). Diversity and dissimilarity coefficients: A unified approach. Theor. Popul. Biol..

[56] Redhead JW, Dreier S, Bourke AF, Heard MS, Jordan WC, Sumner S, Wang J, Carvell C (2016). Effects of habitat composition and landscape structure on worker foraging distances of five bumble bee species. Ecol. Appl..

[57] Ricotta C (2005). Additive partitioning of Rao’s quadratic diversity: A hierarchical approach. Ecol. Model..

[58] Ricotta C, Pavoine S, Bacaro G, Acosta AT (2012). Functional rarefaction for species abundance data. Methods Ecol. Evol..

[59] Ricotta C, Szeidl L (2006). Towards a unifying approach to diversity measures: Bridging the gap between the Shannon entropy and Rao’s quadratic index. Theor. Popul. Biol..

[60] Rocchini D (2007). Effects of spatial and spectral resolution in estimating ecosystem *α*-diversity by satellite imagery. Remote Sens. Environ..

[61] Rocchini D, Chiarucci A, Loiselle SA (2004). Testing the spectral variation hypothesis by using satellite multispectral images. Acta Oecologica.

[62] Rocchini D, Marcantonio M, Ricotta C (2017). Measuring Rao’s q diversity index from remote sensing: An open source solution. Ecol. Ind..

[63] Rocchini D, Santos MJ, Ustin SL, Féret J-B, Asner GP, Beierkuhnlein C, Dalponte M, Feilhauer H, Foody GM, Geller GN (2022). The spectral species concept in living color. J. Geophys. Res. Biogeosci..

[64] Rocchini D, Thouverai E, Marcantonio M, Iannacito M, Da Re D, Torresani M, Bacaro G, Bazzichetto M, Bernardi A, Foody GM (2021). rasterdiv-an information theory tailored r package for measuring ecosystem heterogeneity from space: To the origin and back. Methods Ecol. Evol..

[65] Rocchini D, Torresani M, Beierkuhnlein C, Feoli E, Foody GM, Lenoir J, Malavasi M, Moudrỳ V, Šímová P, Ricotta C (2022). Double down on remote sensing for biodiversity estimation: A biological mindset. Commun. Ecol..

[66] Rossi C, Kneubühler M, Schütz M, Schaepman ME, Haller RM, Risch AC (2022). Spatial resolution, spectral metrics and biomass are key aspects in estimating plant species richness from spectral diversity in species-rich grasslands. Remote Sens. Ecol. Conserv..

[67] Rossignol N, Chadoeuf J, Carrère P, Dumont B (2011). A hierarchical model for analysing the stability of vegetation patterns created by grazing in temperate pastures. Appl. Veg. Sci..

[68] Roussel J-R, Auty D, Coops NC, Tompalski P, Goodbody TR, Meador AS, Bourdon J-F, De Boissieu F, Achim A (2020). lidr: An r package for analysis of airborne laser scanning (ALS) data. Remote Sens. Environ..

[69] Saunders DA, Hobbs RJ, Margules CR (1991). Biological consequences of ecosystem fragmentation: A review. Conserv. Biol..

[70] Scheper J, Bommarco R, Holzschuh A, Potts SG, Riedinger V, Roberts SP, Rundlöf M, Smith HG, Steffan-Dewenter I, Wickens JB (2015). Local and landscape-level floral resources explain effects of wildflower strips on wild bees across four European countries. J. Appl. Ecol..

[71] Scheper J, Reemer M, van Kats R, Ozinga WA, van der Linden GT, Schaminée JH, Siepel H, Kleijn D (2014). Museum specimens reveal loss of pollen host plants as key factor driving wild bee decline in The Netherlands. Proc. Natl. Acad. Sci..

[72] Tamburlin D, Torresani M, Tomelleri E, Tonon G, Rocchini D (2021). Testing the height variation hypothesis with the R Rasterdiv package for tree species diversity estimation. Remote Sensing.

[73] ten Harkel J, Bartholomeus H, Kooistra L (2019). Biomass and crop height estimation of different crops using UAV-based lidar. Remote Sens..

[74] Thessen A (2016). Adoption of machine learning techniques in ecology and earth science. One Ecosyst..

[75] Thouverai E, Marcantonio M, Lenoir J, Galfré M, Marchetto E, Bacaro G, Gatti RC, Da Re D, Di Musciano M, Furrer R (2023). Integrals of life: Tracking ecosystem spatial heterogeneity from space through the area under the curve of the parametric Rao’s q index. Ecol. Complex..

[76] Titeux N, Brotons L, Settele J (2019). Ipbes promotes integration of multiple threats to biodiversity. Trends Ecol. Evol..

[77] Torresani M, Kleijn D, de Vries JPR, Bartholomeus H, Chieffallo L, Gatti RC, Moudrỳ V, Da Re D, Tomelleri E, Rocchini D (2023). A novel approach for surveying flowers as a proxy for bee pollinators using drone images. Ecol. Ind..

[78] Torresani M, Masiello G, Vendrame N, Gerosa G, Falocchi M, Tomelleri E, Serio C, Rocchini D, Zardi D (2022). Correlation analysis of evapotranspiration, emissivity contrast and water deficit indices: A case study in four eddy covariance sites in italy with different environmental habitats. Land.

[79] Torresani M, Rocchini D, Alberti A, Moudrỳ V, Heym M, Thouverai E, Kacic P, Tomelleri E (2023). Lidar Gedi derived tree canopy height heterogeneity reveals patterns of biodiversity in forest ecosystems. Eco. Inform..

[80] Torresani M, Rocchini D, Sonnenschein R, Zebisch M, Hauffe HC, Heym M, Pretzsch H, Tonon G (2020). Height variation hypothesis: A new approach for estimating forest species diversity with chm lidar data. Ecol. Ind..

[81] Turner W, Spector S, Gardiner N, Fladeland M, Sterling E, Steininger M (2003). Remote sensing for biodiversity science and conservation. Trends Ecol. Evolut..

[82] Wang R, Gamon JA (2019). Remote sensing of terrestrial plant biodiversity. Remote Sens. Environ..

[83] Westphal C, Bommarco R, Carré G, Lamborn E, Morison N, Petanidou T, Potts SG, Roberts SP, Szentgyörgyi H, Tscheulin T (2008). Measuring bee diversity in different European habitats and biogeographical regions. Ecol. Monogr..

[84] Winfree R, Aguilar R, Vázquez DP, LeBuhn G, Aizen MA (2009). A meta-analysis of bees’ responses to anthropogenic disturbance. Ecology.

[85] Wood DJ, Preston TM, Powell S, Stoy PC (2022). Multiple UAV flights across the growing season can characterize fine scale phenological heterogeneity within and among vegetation functional groups. Remote Sens..

[86] Xiang M, Wu J, Wu J, Guo Y, Lha D, Pan Y, Zhang X (2021). Heavy grazing altered the biodiversity-productivity relationship of alpine grasslands in Lhasa River Valley, Tibet. Front. Ecol. Evol..

